# Prospects for biological control of wood-boring insects using phoretic mites

**DOI:** 10.3389/fpls.2026.1805145

**Published:** 2026-04-13

**Authors:** Marielle M. Berto, Daniel Carrillo

**Affiliations:** Tropical Research and Education Center, Department of Entomology and Nematology, University of Florida, Homestead, FL, United States

**Keywords:** cryptic behavior, entomopathogens, phoretic mites, vectors, wood boring insects

## Abstract

A novel biological control strategy using phoretic mites to target wood-boring pest insects is proposed. Phoretic mites are intimately associated with wood-boring insects and can reach habitats inaccessible to standard chemical and biological control strategies. Phoretic mites can deliver microbial biocontrol agents directly to their breeding sites inside tree trunks. These opportunistic mites have diverse feeding habits and behaviors that can be explored for the development of novel biological control strategies. Attributes of different groups of phoretic mites that could make them efficient vectors of microbial agents are discussed. The choice of microbial agents, mass-rearing techniques and possible field-delivery methods for phoretic mites are also discussed.

## 1 Introduction

**Figure 1:**
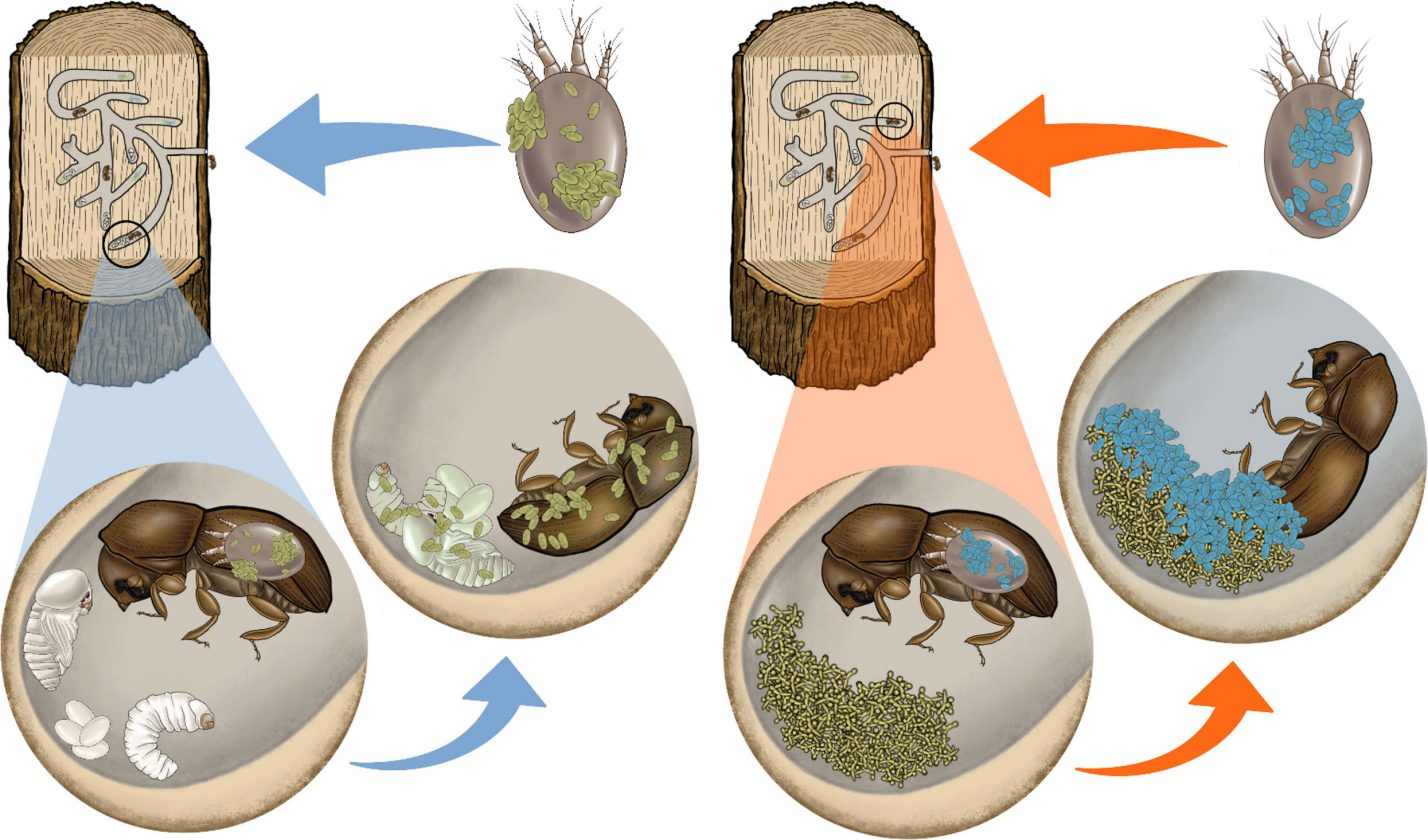
Multitrophic interaction between ambrosia beetles, symbiotic fungi, phoretic mites and beneficial fungal agents. Left diagram: phoretic mites carrying entomopathogenic fungal spores (green) externally. The mites are released in the field, enter the galleries and interact with their phoretic hosts (beetles), infecting the adults and their brood with entomopathogenic fungi. Right diagram: phoretic mites carrying antagonistic fungal spores (blue) externally. The mites are released in the field, enter the galleries and interact with their phoretic hosts (beetles), infecting their nutritional fungal gardens with antagonistic fungi. These fungal agents disrupt the symbiosis between the beetle and their nutritional fungi.

This perspective article proposes a novel biological control strategy using phoretic mites to vector entomopathogenic microbial agents into cryptic breeding sites of wood-boring pests (**Figure 1**). Phoretic mites are intimately associated with wood-boring insects. They can deliver microbial biocontrol agents directly to their breeding sites within tree trunks, representing a significant improvement over the poor control achieved by spraying conventional insecticides or microbial agents on external parts of trees (Carrillo et al., 2013, Carrillo et al., 2015). These opportunistic mites have diverse feeding habits and behaviors that can be explored for the development of novel biological control strategies. Attributes of different groups of phoretic mites that could make them efficient vectors of microbial agents are discussed, as well as the selection of microbial agents, mass-rearing techniques, and possible field-delivery methods for phoretic mites.

## 2 Occurrence and diversity of phoresy in mites

Phoretic mites use insects for dispersal and are physically attached to their host during at least one stage of their life cycle. They often cease feeding during transportation and have mild to extreme morphological adaptations for strong attachment to their insect carrier. These modifications suggest that phoresy is part of a long-term coevolutionary process between the mites and their carriers (Seeman and Walter, 2023). Phoretic mites usually have high reproductive rates, short life cycles and rapid immature development (r-strategists). They often occupy ephemeral habitats, such as carrion, dung, animal nests, insect galleries and decomposing organic matter (Perotti and Braig, 2009; Silva et al., 2018). These mites tend to deplete resources quickly and need to find new habitats quickly as well. However, they lack wings and, because of their minute size, they cannot reach new areas with resources solely by crawling. Many mite groups resort to wind for transportation, while others ride on larger organisms like insects, birds, mammals and other mites (Niogret et al., 2006; Siepel, 1994).

Phoretic mites are diverse in their behavior, physiology, host specificity and degrees of adaptation for phoresy. Most phoretic mites are found within the mite taxons Mesostimata, Astigmata and Prostigmata. Mesostigmatid mites are usually predatory, parasitic, and/or fungivorous. Phoretic mesostimatids in general have no prominent morphological modifications, but rather adaptations such as the presence of claws, reduction or enlargement of particular setae, and chelicerae modifications (Krantz, 1998; Evans and Sheals, 1959; Baker and Delfinado-Baker, 1985; Delfinado-Baker et al., 1984). The most noticeable adaptations happen in the superfamilies Uropodoidea and Sejoidea. Deutonymphs of phoretic species in these groups are more tolerant to desiccation and produce a pedicel to attach to their host. This structure is secreted by a pedicellar gland located around the anal portion of the mites and hardens when exposed to the air (Bajerlein and Witaliński, 2012). Approximately 60% of superfamilies within the Mesostigmata are estimated to engage in phoresy (Seeman and Walter, 2023).

Astigmatid mites are usually parasites of vertebrates, fungivores, detritivores or pests of stored food and bulbs. Adults, larvae and protonymphs of Astigmata are generally soft bodied with very little sclerotization. The phoretic deutonymph, also called hypopus, is a facultative life stage between the protonymph and tritonymph with special adaptations for phoretic transport. Hypopi are flattened, well-sclerotized, with shortened legs, no functional mouthparts and have a range of attachment structures ventrally, such as sucker plates (Binns, 1982). Hypopi are frequently more mobile than other life stages, actively seeking a host. However, some hypopi are immobile, cyst-like, and resemble a diapause state in insects (Wallace, 1960). Other life stages of the Astigmata are rarely phoretic. Within the family Acaridae the genus *Histiogaster* is frequently found in phoretic associations with many insect hosts, especially on beetles (Klimov et al., 2022; Berto et al., 2025a). Hypopus formation in this genus is common and triggered by changes in abiotic factors such as temperature and humidity.

Phoresy in the Prostigmata is particularly common in the cohort Heterostigmata. The associations of Heteristigmatid mites with insects vary from facultative to obligate phoresy to parasitism (Kaliszewski et al., 1995). Some are highly host-specific and attach to specific sites on the host body, such as beneath a beetle’s elytra or between the coxae in the base of their legs. The families Pygmephoridae, Neopygmephoridae, Scutacaridae, and Pyemotidae, which are mainly fungivorous and arthropod parasites that often reside in nests of social insects (Khaustov et al., 2022). They often exhibit phoretic polymorphism in adults (Walter and Proctor, 2013; Baumann, 2018; Katlav et al., 2020). The presence of enlarged claws and robust anterior legs is a common feature in phoretic females that helps them firmly attach to their host during phoretic dispersal (Ebermann, 1991; Camerik et al., 2006).

Phoretic mites of wood-boring insects usually live in the same habitat as their hosts. Some appear to be more habitat-specific than host-specific, occurring on many tree species and associated with different host insects. Some mites occasionally engage in phoresy by accidentally encountering and utilizing a host for transportation (Masán, 1993; Pfammatter et al., 2015; 2013), while others actively seek a host when conditions are not favorable to them or their progeny. A species that represents these facultative phoretic associations is *Histiogaster arborsignis* (Acari: Acaridae), a cosmopolitan phoretic mite reported in association with more than 15 insect species across the orders Diptera, Hymenoptera and mainly Coleoptera (O’Connor, 1990). They are not linked to a specific insect host but rather to several microorganisms across woody habitats worldwide (**Table 1**). Phoretic mites adapted to woody habitats, such as *H. arborsignis*, could disseminate microbial agents within a tree even in the absence of the target pest, enabling both preventive and curative treatments. Additionally, they could simultaneously target multiple wood-boring pests infesting a tree.

**Table 1:** Insect, plant and fungal hosts reported for *Histiogaster arborsignis* (Acari: Acaridae). NA= not available.

Insect host	Plant host	Fungal host	Locality	References
Coleoptera				
Buprestidae				
*Buprestis striata*	NA	NA	MI, USA	O’Connor (1991)
*Chalcophora* sp.	NA	NA	MI, USA	O’Connor (1991)
Cerambycidae				
*Acmaeops proteus*	NA	NA	MI, USA	O’Connor (1991)
*Monochamus carolinensis*	*Pinus taeda*	NA	NA	Kinn (1989)
*Monochamus scutellatus*	NA	NA	MI, USA	O’Connor (1991)
*Monochamus titillator*	*Pinus taeda*	NA	LA, USA	Kinn (1989) Kinn (1987)
*Neacanthosinus obsoletus*	*Pinus taeda*	NA	LA, USA	Kinn (1989); Kinn (1987)
*Physocnemum brevilineum*	NA	NA	MI, USA	O’Connor (1991)
*Xyloterus sagittatus*	*Pinus taeda*	NA	NA	Kinn (1989)
Cleridae				
*Enoclerus lecontei*	*Pinus ponderosa Pinus contorta*	NA	CO, USA	Mercado (2015)
*Enoclerus nigrifrons*	*Pinus resinosa*	NA	WI, USA	Pfammatter et al. (2016)
*Enoclerus nigripes*	NA	NA	NJ, USA	O’Connor (1991)
*Enoclerus sphegeus*	*Pinus ponderosa*	NA	AZ, USA	Mercado (2015); Hofstetter et al. (2013)
*Thanasimus dubius*	*Pinus ponderosa*	NA	WI, USA	Pfammatter et al. (2016)
*Thanasimus undatulus*	*Pinus ponderosa* & *Pinus contorta*	NA		Mercado (2015)
Cucujidae				
*Silvanus unidentatus*	NA	NA	Poland	Chmielewski (1977)
Curculionidae				
*Ambrosiodmus lecontei*	*Persea americana*	NA	FL, USA	Berto et al. (2025a)
*Anthonomus* sp.	*Pinus ponderosa*	NA	WI, USA	Pfammatter et al. (2016)
*Dendroctonus frontalis*	*Pinus taeda* & *Pinus oocarpus*	NA	TX, LA, AK, USA; Mexico, Honduras, Guatemala	Woodring (1963); Moser and Cross (1975) Kinn (1979); Moser and Roton (1971); Kinn (1979); Hofstetter et al. (2013); Woodring (1966); Moser et al. (1974); Woodring (1966)
*Dendroctonus mexicanus*	*Pinus leiophylla*	NA	Mexico	Woodring (1966)
*Dendroctonus ponderosae*	*Pinus ponderosa* & *P. contorta*	NA	CO & SD, USA Canada	Moser et al., 1971 Mori et al. (2011) Mercado (2015); Woodring (1966)
*Dendroctonus rufipennis*	*Picea* spp.	NA	AK, USA	Cardoza et al. (2008)
*Dendroctonus terebrans*	*Pinus taeda*	NA	LA, USA	Woodring (1966); Moser and Roton (1971)
*Dendroctonus valens*	*Pinus ponderosa*	NA	AZ & WI, USA	Pfammatter et al. (2016); Hofstetter et al. (2013); Pfammatter (2015)
*Dendroctonus* sp.	*Pinus jeffreyi*	NA	CA, USA	Woodring (1966)
*Hylastes porculus*	*Pinus taeda*	NA	GA, USA	Pfammatter et al. (2016); Pfammatter (2015)
*Hylurgopinus rufipes*	NA	NA	OH, USA	Woodring (1966)
*Ips avulsus*	*Pinus echinata* & *Pinus taeda*	NA	GA, LA & NC, USA	Pfammatter et al. (2016); Pfammatter (2015); Woodring (1966)
*Ips calligraphus*	*Pinus, echinata*, *Pinus ponderosa* & *Pinus taeda*	NA	AZ, GA & LA,USA	Pfammatter et al. (2016); Woodring (1966); Moser and Roton (1971)
*Ips grandicollis*	*Pinus resinosa & Pinus taeda*	NA	GA & WI, USA	Pfammatter et al. (2016); Pfammatter and Raffa (2015); Pfammatter (2015); Woodring (1966); Moser and Roton (1971)
*Ips guildi*	*Pinus* sp.	NA	Canada	Woodring (1966)
*Ips knausi*	*Pinus ponderosa*	NA	CO, USA	Moser et al., 1971
*Ips mexicanus*	*Pinus lutea*	NA	Mexico	Woodring (1966)
*Ips pini*	*Pinus banksiana*, *Pinus ponderosa* & *Pinus resinosa*	NA	AZ, CO, GA, MI & WI, USA	O’Connor (1991); Pfammatter et al. (2016); Pfammatter (2015); Woodring (1966)
*Ips platygaster*	*Pinus radiak*	NA	CA, USA	Woodring (1966)
*Ips radiak*	*Pinus radiak*	NA	CA, USA	Woodring (1966)
*Ips typographus*	NA	NA	Germany	Moser and Bogenschütz (1984)
*Ips* sp.	*Pinus taeda* & *Pinus oocarpus*	NA	LA, USA; Honduras	Woodring (1966)
*Orthotomicus caelatus*	*Pinus strobus*	NA	Canada	Woodring (1966)
*Xyleborinus saxesenii*	*Persea americana*	NA	FL, USA	Berto et al. (2025a)
*Xyleborus affinis*	*Persea americana*	NA	FL, USA	Berto et al. (2025a)
*Xyleborus bispinatus*	*Persea americana*	NA	FL, USA	Berto et al. (2025a)
*Xyloterinus politus*	NA	NA	NY, USA	O’Connor (1991)
Nitidulidae				
*Glischrochilus quadrisignatus*	NA	NA	NY, USA	O’Connor (1991)
Ostomatidae				
*Grynocharis quadrilineata*	NA	*Pleurotis ostreatus*	MI, USA	O’Connor (1991)
Pythidae				
*Pytho americanus*	NA	NA	MI, USA	O’Connor (1991)
Silphidae				
*Nicrophorus orbicollis*	NA	NA	MI, USA	O’Connor (1991)
Silvanidae				
*Silvanus unidentatus*	NA	NA	Poland	Chmielewski (1977)
Trogossitidae				
*Temnoscheila chlorodia*	NA	NA	AZ, USA	Hofstetter et al. (2013)
Hymenoptera				
Braconidae				
*Atanycolous ulmicola*	NA	NA	MI, USA	O’Connor (1991)
Ichneumonidae				
*Dolichomitus imperator*	NA	NA	MI, USA	O’Connor (1991)
*Dolichomitus irratator*	NA	NA	Canada	O’Connor (1991)
*Dolichomitus tuberculatus*	NA	NA	NY, USA	O’Connor (1991)
*Megarhyssa macrurus*	NA	NA	MI, USA	O’Connor (1991)
*Neoxorides pillulus*	NA	NA	MI, USA	O’Connor (1991)
*Xylophylax macrocephala*	NA	NA	MI, USA	O’Connor (1991)
Siricidae				
*Sirex* sp.	*Abies* sp.	NA	Canada	Woodring (1966)
Diptera				
Asilidae				
*Laphria janus*	NA	NA	MI, USA	O’Connor (1991)
*Laphria posticata*	NA	NA	MI, USA	O’Connor (1991)
Syrphidae				
*Somula decora*	NA	NA	MI, USA	O’Connor (1991)
	*Acer saccharum*	NA	MI, USA; Canada	O’Connor (1991)
	*Pinus resinosa*	NA	MI, USA	O’Connor (1991)
	*Betula alleghaniensis*	NA	MI, USA	O’Connor (1991)
	*Betula papyrifera*	*Piptoporus betulinus*	MI, USA	O’Connor (1991)
	*Bertholletia excelsa*	NA	Brazil	Woodring (1966)
	*Acer saccharum*	*Pleurotus ostreatus*	MI, USA	O’Connor (1991)
		*Phellinus gilvus*	IL, USA	
		*Coriolus versicolor*	MI, USA	
	*Fagus grandifolia*	*Fomes fomentarius*	NY, USA	O’Connor (1991)
	*Pinus monticola*	*Cronartium ribicola*	ID, USA	Furniss et al. (1972)
	*Solanum lycopersicum*	–	Mexico	Woodring (1966)
	*Albizia lebbeck*	–	Cuba	De la Torre Santana (2013)

The foraging behavior of arthropods used as vectors of microbial agents is critical. Many phoretic mites of wood-boring insects have free-living stages that develop on the tree and modified life stages dedicated to phoresy. The modified stages, also called phoretomorphs, are usually an immature stage (i.e., deutonymph) or the adult female that displays strong host-seeking behavior (Moser and Cross, 1975; Wallace, 1960). Host-seeking behavior varies greatly among mite groups, and little is known about their ecology and behavior. Research effort in this area is necessary if phoretic forms are to be used as vectors of microbial agents.

Mites engage in phoresy to find new resources and habitats for reproduction. Therefore, environmental conditions are most likely the main inducer of phoresy in mites. Temperature, humidity, light, overcrowding, lack of food, food quality and deterioration are common inducers of phoresy in mites (Griffiths, 1966; Polezhaev, 1938; Knülle, 1991; Athias-Binche, 1995). Besides abiotic factors, mites exhibit genotypic differences that determine the propensity for phoresy and the formation of phoretomorphs (Knülle, 1987; Athias-Binche, 1993; Knülle, 2003; Corente and Knülle, 2003). Phoretomorphs are usually several times more resistant to fluctuating environmental conditions than other life stages. Phoretic deutonymphs of the Astigmata (hypopi) produce a thicker and more sclerotized integument that grants them a certain degree of tolerance against changes in temperature and relative humidity (Wallace, 1960; Knülle, 1995). Additionally, phoretomorphs develop structures, such as claws, ventral suckers, and pedicels, that allow them to remain firmly affixed to the host.

## 3 Ability of phoretic mites to acquire and disseminate beneficial microbial agents

### 3.1 Natural association between mites and microorganisms

Fungivory is widely documented across taxa within the Astigmata. For instance, different *Histiogaster* species have reported to feed on an array of fungal taxa (Okabe, 1992, Okabe, 1993; Woodring, 1966; Berto et al., 2025a). Oribatid mites play an important role as decomposers in organic matter-rich habitats, such as soil, leaf litter, and plants, and are also closely associated with fungi (Maraun et al., 1998; Schneider et al., 2005). Due to their saprophytic feeding habits, oribatids also associate with bacterial communities that facilitate enzymatic digestion of their food (Smrž, 2010). Their closeness to microbe-rich environments highlights their potential to vector not only fungi, but also other microorganisms, including entomopathogenic bacteria that may be harmful to insect pests. Although these mites are generally slow-moving and rarely considered in biocontrol applications (Ahadiyat and Akrami, 2015; Knee and Beaulieu, 2013), some species are phoretic on beetles, and their potential as vectors of microbial agents should be assessed.

### 3.2 Ability of mites to transport fungi

Heterostigmatid mites are also fungivorous and have evolved specialized structures for transporting fungal spores. Ebermann and Hall (2003) reported the presence of sporothecae in phoretic mites of the family Scutacaridae, specifically *Imparipes haeseleri* and *I. apicola*. These species are phoretic on wood and soil-dwelling Hymenoptera, and the spores they carry are likely associated with nests of their phoretic hosts. Sporotheca have also been recorded in other mite groups within the Heterostigmata, including the Siteroptiidae and Tarsonemidae (Laemmlen and Hall, 1973; Moser, 1985). Some mites in the large genus *Tarsonemus* (Heterostigmata) are phoretic on the southern pine beetle, *Dendroctonus frontalis* (Curculionidae: Scolytinae) (Moser, 1985). These mites can disseminate a blue stain fungus, *Ophiostoma minus* (Ophiostomatales: Ophiostomataceae) (Lombardero et al., 2000).

Mites do not need to have a sporothecae to transport fungi. Many fungivorous, detritivorous and predatory mites acquire fungal spores incidentally during foraging and feeding. Fungivores and detritivores typically acquire fungal propagules directly from their food sources and surrounding substrates, while predators may ingest fungal material indirectly through their prey. Fungal spores have been recovered from gut contents of several mite groups, including Mesostigmata, Astigmata, Prostigmata and are particularly frequent in Oribatida (Hofstetter and Moser, 2014; Walter, 1988; Gong et al., 2018).

Perhaps the most common way mites transport fungi is externally, on their cuticle. Cracks and crevices are perfect microhabitats for mites and microorganisms. Therefore, many mite groups are in constant contact with fungal structures that attach to their body. Moser et al. (1989) identified ten different ascospores carried externally by distinct groups of phoretic mites associated with the European spruce bark beetle, *Ips typographus* (Coleoptera: Curculionidae). Some of the spores were already germinating, suggesting a phoretic association between the mites and the fungi. Similarly, Ocak et al. (2008) reported 19 fungal species recovered from the surface of various oribatid mites. A broad range of fungal species can be carried externally by mites. These associations need to be explored in pest management applications.

### 3.3 Considerations for the selection of microbial agents vectored by phoretic mites

Suitable microbial agents for biocontrol applications using phoretic mites would cause mortality in the target pest without significantly harming the mite vector. If the microbial agent occurs naturally in the environment and/or is part of the vector’s nutrition, it is probably more adequate for biocontrol, and could potentially be mass-cultured in conjunction with the mite vector.

Fungi are the most studied group of microbes as potential controllers of wood-boring pests. *Metarrhizium anisopliae* and *Beauveria bassiana* have proven effective when in contact with wood-boring insects (Hajek and Bauer, 2009). Phoretic mites could assist in vectoring such species into insect galleries; however, little is known about the pathogenicity of these fungi to phoretic mites. Schabel (1982) assessed two phoretic mites as vectors of *M. anisopliae* targeting the pales weevil, *Hylobius pales* (Coleoptera: Curculionidae), a serious pine forest pest. *Macrocheles* sp. (Mesostigmata: Macrochelidae) and *Histiogaster anops* (Astigmata: Acaridae) were infected by *M. anisopliae*. However, *Macrocheles* sp. survived significantly longer and disseminated more *M. anisopliae* spores, causing 80% of beetle mortality. By contrast, *H. anops* survived for about 3 days after fungal inoculation and was unable to induce significant beetle mortality. These results indicated that vectors don’t need to be completely resistant to the microbial agent, but must survive long enough to transmit sufficient pathogen propagules to the target pest.

Besides entomopathogenic fungi, potential antagonists of wood-boring insect symbionts, such as *Trichoderma* spp. could be used in biocontrol applications using phoretic mites (**Figure 1**). These microbes could indirectly harm the pest insects by degrading their food and habitat and/or by disrupting their symbiotic associations with other microorganisms. Along these lines, reports of the generalist fungivore *Histiogaster* sp. being collected and reared on *T. harzianum* (Hiraoka et al., 2002; Okabe, 1993, Okabe, 1992) are encouraging and suggest their potential combination in biocontrol applications targeting wood-boring pests that practice fungiculture, such as ambrosia beetles.

Some phoretic mite species can be easily mass-reared. For fungivorous mites, such as *H. arborsignis* and other astigmatid mites, it is possible to rear both vectors and microbial agents within the same system (Berto et al., 2025b). Astigmatid mites are common pests of stored grains and can be mass reared in different substrates, such as brewer’s yeast, wheat bran, rice bran, flour and barley (Hubert et al., 2013; Aratchige et al., 2010; Ballal et al., 2021; Berto et al., 2025b). Some entomopathogenic fungi, such as *Beauveria* and *Metarhizium* species, can be mass-produced through solid-substrate fermentation using the same substrate (Nelson et al., 1996; Jaronski, 2013). Little research is available on this matter; however, Okabe (1992, Okabe, 1993), O’Connor (1990), and Berto et al. (2025a) showed that astigmatid mites can be reared solely on fungal species, including beneficial fungi. For instance, some authors reported collecting and maintaining a colony of *Histiogaster* sp. on the beneficial fungus *T. harzianum* (Hiraoka et al., 2002; Okabe, 1993, Okabe, 1992).

### 3.4 Delivery methods

Delivery methods for augmentative or inundative releases of predatory mites in the family Phytoseiidae have been developed (McMurtry et al., 2015). Sachets that facilitate the slow release of predators and protect both the predators and their rearing substrate from adverse abiotic factors have been widely used in biological control programs (Shimoda et al., 2017). An adaptation of such sheltered devices deployed on tree trunks could facilitate the ambulatory dispersal of phoretic mites and protect the microbial agent. In addition, fiber bands placed around tree trunks containing *M. anisopliae* and *Beauveria brongniartii* were developed to control wood boring pests (Hajek et al., 2006). The fiber bands significantly increase the viability of entomopathogens infecting crawling insects on tree bark. Phoretic mites could benefit from the shelter offered by the bands, while aiding the transport of beneficial fungi to breeding sites of wood-boring pests within the tree trunks. In addition, conventional spray applications directed at the tree trunk could be more cost-effective when labor costs and availability are limiting factors.

## 4 Safety of using phoretic mites as vectors of microbial agents

Augmenting populations of phoretic mites may pose risks to non-target insects and their associated microbial communities. Such effects are particularly concerning for organisms involved in wood decomposition, a fundamental ecological process that contributes to global biogeochemical cycles and represents a major carbon pool in forest ecosystems (Benbow et al., 2019; Pan et al., 2011). Several taxa, including termites and large cerambycid and passalid beetles, influence wood decomposition not only directly through the consumption and fragmentation of deadwood, but also indirectly through interactions with microbial communities, particularly fungi and bacteria. Releasing phoretic mites carrying specific microbes could potentially disrupt these organisms and processes that drive wood decomposition. For this reason, such releases should be conducted cautiously and limited to trees infested by wood-boring pests. Consequently, the proposed biocontrol strategy will likely require prior monitoring to determine the distribution of the target wood-boring pest. Monitoring tools such as lures or traps may be necessary to guide the targeted deployment of phoretic mites, restricting releases to trees already infested or at high risk of infestation. Another key consideration is selecting microbial agents that do not interfere with the microorganisms involved in natural wood decomposition. From a regulatory standpoint, taxonomic knowledge of the mite species used will also be important, particularly whether the species is already present in the country or region where releases are planned. Cosmopolitan species, such as H. arborsignis (**Table 1**), may be more acceptable due to their widespread distribution in wooded habitats worldwide. However, mite species with more specific host associations could also be considered, as they may present a lower risk of non-target effects. Evaluating the ecological implications of using phoretic mites as vectors of microbial agents is essential to ensure a balanced assessment of this emerging pest management strategy.

## 5 Ecological context and applicability

The applicability of the proposed management strategy will largely depend on the ecology of the wood-boring species and the characteristics of the ecosystem in which it occurs. Mites could be deployed to suppress infestation hotspots in areas where environmental conditions are suitable for their survival and establishment. Intensively managed systems, where pest infestations are routinely monitored and spatially delimited, may provide an appropriate context for targeted mite releases. In contrast, implementation may be more challenging in landscapes where wood-boring pests are randomly distributed across large areas. Furthermore, the selected mite species must be capable of surviving, dispersing and colonizing the same environmental conditions and microhabitats occupied by the target wood boring pest. Potential targets include invasive beetles in the families Buprestidae, Cerambycidae, Bostrichidae, and Curculionidae, which are common pests of native forests and agricultural ecosystems (Haack, 2001; 2006; FAO, 2009; Carrillo et al., 2012; Mankin et al., 2018). The Emerald Ash Borer, *Agrilus planipennis* (Coleoptera: Buprestidae) bores galleries in Ash trees and may go undetected for years until the adults emerge (Cappaert et al., 2005). The Asian Longhorned Beetle, *Anoplophora glabripennis* (Coleoptera: Cerambycidae), and the Citrus Longhorned Beetle, *A. chinensis*, are highly polyphagous wood-borers that threaten tree ecosystems in Europe and North America (Haack et al., 2010). Other wood-boring insects cause damage indirectly by inoculating plants with aggressive pathogens. Ambrosia beetles (Coleoptera: Curculionidae: Scolytinae) have recently emerged as vectors of plant pathogens, including *Harringtonia lauricola* and *Fusarium* spp (Carrillo et al., 2014, Carrillo et al., 2016). In addition, sawflies (Hymeptera: Cephidae) can damage forest trees and agricultural crops (Knodel et al., 2010). These are a few examples of wood-boring pests that could potentially be controlled by microbial control agents delivered by phoretic mites.

## 6 Conclusions

Current integrated pest management strategies are insufficient to effectively control wood-boring insects. Mites are tiny organisms with diverse feeding habits and behaviors, able to reach breeding sites of many challenging pests. Phoretic mites exhibit diverse adaptations for phoresy and host specificity. Many groups present phoretomorphs dedicated to dispersal. These morphs are usually more resistant to environmental factors and may be able to overcome harsh temperatures and drought. Many phoretic mites are fungivores and form strong associations with a wide array of fungal species in their environments. Mite species within the Astigmata, Oribatida and Heterostigmata carry and inoculate fungal spores into other environments, sometimes affecting their phoretic hosts. Microbial agents carried by phoretic mites can range from entomopathogens (i.e., *B. bassiana* and *M. anisopliae*) to fungal competitors (i.e., *T. harzianum*) that can antagonize with the food sources of their phoretic hosts. Natural associations of phoretic mites and fungi make it possible to rear microbial agents and their vectors together.

Significant knowledge gaps must be addressed before the proposed use of phoretic mites in biocontrol can be implemented. Efficient delivery methods and mass rearing protocols for both microbial agents and vectors are still under development (Berto et al., 2025a, Berto et al., 2025b). Controlled experiments are needed to better understand the foraging and host-seeking behaviors of phoretic mite species. In addition, the efficacy of different phoretic mites and delivery methods should be evaluated under a range of conditions and against different wood-boring pests. Such evaluations should begin with small proof-of-concept cage experiments and progress to open-field trials under realistic conditions. Further research will also be necessary to determine how this approach can be integrated into existing integrated pest management programs targeting wood-boring pests. Despite these uncertainties, phoretic mites represent a promising and innovative integrated pest management strategy for managing pests that are difficult to control using conventional methods.

## Data Availability

The original contributions presented in the study are included in the article/supplementary material. Further inquiries can be directed to the corresponding authors.
